# Significance of Cancer-Associated Fibroblasts in the Interactions of Cancer Cells with the Tumor Microenvironment of Heterogeneous Tumor Tissue

**DOI:** 10.3390/cancers15092536

**Published:** 2023-04-28

**Authors:** Yoshimi Arima, Satoko Matsueda, Hideyuki Saya

**Affiliations:** Cancer Center, Fujita Health University, Toyoake 470-1192, Japan

**Keywords:** cancer-associated fibroblast, tumor microenvironment, desmoplastic stroma, tumor heterogeneity, immune suppression, immunocompetent mouse model, immunotherapy

## Abstract

**Simple Summary:**

The tumor microenvironment (TME) is composed of various cell types and extracellular components and plays an important role in cancer development and progression, as well as in therapeutic resistance and metastasis. Cancer-associated fibroblasts (CAFs) are a key cell type in the TME and interact with cancer cells as well as with other TME cells, such as immune cells. Immunocompetent mouse cancer models that recapitulate the interactions of cancer cells with the TME have provided a better understanding of the TME network and support the development of new therapeutic strategies. In this review, we focus on cancer cell–TME interactions in heterogeneous tumor tissue, and we provide an overview of therapeutic strategies that target the TME.

**Abstract:**

The tumor microenvironment (TME) plays a key role in cancer development and progression, as well as contributes to the therapeutic resistance and metastasis of cancer cells. The TME is heterogeneous and consists of multiple cell types, including cancer-associated fibroblasts (CAFs), endothelial cells, and immune cells, as well as various extracellular components. Recent studies have revealed cross talk between cancer cells and CAFs as well as between CAFs and other TME cells, including immune cells. Signaling by transforming growth factor-β, derived from CAFs, has recently been shown to induce remodeling of tumor tissue, including the promotion of angiogenesis and immune cell recruitment. Immunocompetent mouse cancer models that recapitulate interactions of cancer cells with the TME have provided insight into the TME network and support the development of new anticancer therapeutic strategies. Recent studies based on such models have revealed that the antitumor action of molecularly targeted agents is mediated in part by effects on the tumor immune environment. In this review, we focus on cancer cell–TME interactions in heterogeneous tumor tissue, and we provide an overview of the basis for anticancer therapeutic strategies that target the TME, including immunotherapy.

## 1. Introduction

Research conducted over the past several decades has examined the characteristics of cancer, the molecular mechanisms underlying carcinogenesis, and the development of new cancer treatments. Despite this effort, cancer remains one of the leading causes of death worldwide. Cancer is invasive within the tissues and organs of its development, is able to metastasize to other sites, and can recur after its removal or treatment. Recent studies have revealed that the tumor microenvironment (TME) plays a central role in cancer development and progression as well as in its therapeutic resistance, metastasis, and recurrence in clinical practice [1,2,3]. The TME of most solid cancers is highly complex and heterogeneous. It is composed of various cell types—including fibroblasts, endothelial cells, and immune cells—as well as extracellular factors, such as secreted molecules (including cytokines and growth factors), exosomes, and the extracellular matrix (ECM) [4,5,6]. Cancer-associated fibroblasts (CAFs) are a key component of the TME. Cancer cells interact with CAFs, and CAFs interact with other cells of the TME, with such interactions with immune cells resulting in immune suppression in tumor tissue [7]. A better understanding of the TME is thus likely to provide a basis for the development of new cancer treatments. In this review, we focus on the interactions between cancer cells and the TME within heterogeneous tumor tissue, and we provide an overview of the basis for therapeutic strategies that target the TME, including immunotherapies.

## 2. The TME in Tumor Tissue

### 2.1. Components of the TME

The TME in most solid cancers is complex and shows intratumoral heterogeneity. Its composition varies, but components include tumor stromal cells, such as CAFs [4], the ECM, extracellular vesicles, endothelial cells, and various immune cell types [8,9,10,11,12,13]. Physical and chemical characteristics of the TME—such as a low pH, hypoxia, high interstitial pressure, and fibrosis—are also important determinants of cancer progression [14,15,16].

Natural killer (NK) cells, CD8^+^ cytotoxic T cells, M1 macrophages, T helper 1 cells, and antigen-presenting cells in the TME suppress tumor growth. However, the TME also includes protumoral immune cells—such as neutrophils, tumor-associated macrophages (TAMs), myeloid-derived suppressor cells (MDSCs), and regulatory T cells (Tregs)—that contribute to local immune suppression and thereby promote tumor cell survival through evasion of immune destruction [6,17]. Neutrophils also promote angiogenesis and thereby facilitate metastasis, and they are associated with poor prognosis. Blood vessels in the TME supply oxygen and nutrients that support the growth of tumor tissue, as well as provide a conduit for metastasis to distant sites via the bloodstream. Proangiogenic molecules—such as vascular endothelial growth factor (VEGF), transforming growth factor-α (TGF-α), TGF-β, and epidermal growth factor (EGF)—as well as antiangiogenic molecules, including angiostatin, endostatin, interleukin (IL)–12, thrombospondin-1, tissue inhibitors of metalloproteinases (TIMPs), and interferons, are all well-studied angiogenic regulators [18].

### 2.2. The Tumor Stroma

**Figure 1:**
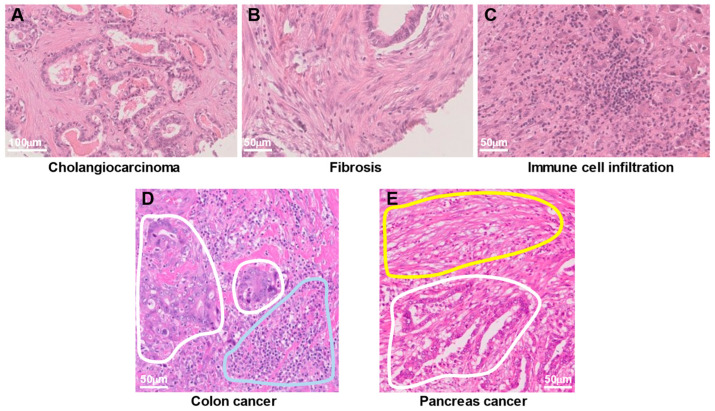
Heterogeneous cell populations in human cancer tissue. (**A**–**C**) Hematoxylin and eosin (H&E) staining of the same cholangiocarcinoma tissue specimen reveals cancer cells (**A**), fibrotic tissue composed of fibroblasts (**B**), and infiltration of immune cells consisting mostly of lymphocytes (**C**). (**D**) H&E staining of colon cancer tissue shows cancer cells (areas surrounded by white lines) in close proximity to infiltrating immune cells (area surrounded by the blue line). (**E**) H&E staining of a pancreatic cancer specimen reveals fibrotic tissue (area surrounded by the yellow line) adjacent to cancer cells (area surrounded by the white line).

The TME and tumor stroma are terms often used synonymously. However, whereas the TME is a non-tumor cell region of tumor tissue, including acellular components from a cell biological point of view, the tumor stroma is a non-tumor cell region of tumor tissue from a pathomorphological point of view. Histopathologic analysis has shown that cancer tissue contains various cell types at high density in addition to cancer cells. Staining of human bile duct cancer (cholangiocarcinoma) with hematoxylin and eosin (H&E), for example, reveals cancer cells (Figure 1A), fibrotic tissue composed of fibroblasts (Figure 1B), and inflammatory cell infiltrates consisting mostly of lymphocytes (Figure 1C). In addition, cancer cells exist in close proximity to immune cells or fibroblasts, as is evident in images of human colon cancer (Figure 1D) and pancreatic cancer (Figure 1E), respectively. Such histopathologic images have revealed that pancreatic cancer and cholangiocarcinoma are the most stroma-rich cancer types, with the dense stroma being thought to contribute to their poor prognosis.

Pancreatic ductal adenocarcinoma (PDAC) is a highly aggressive and lethal disease and is one of the most stroma-rich cancer types, with the stroma accounting for up to 90% of the tumor volume [19]. The dense fibrotic stroma is closely associated with cancer cells, and interactions of PDAC cells with the stroma contribute to tumor growth and progression. In addition to the genetic (such as *KRAS* mutation) and epigenetic alterations of PDAC cells that promote tumorigenesis, the PDAC stroma imposes physical and biological barriers to treatment, including chemotherapy, radiotherapy, targeted therapy, and immunotherapy [20]. In particular, the stromal microenvironment (including CAFs, the ECM, and vasculature) of PDAC is a major obstacle to drug delivery to the tumor bed and plays a key role in therapeutic resistance [1]. Signaling by the integrin–FAK (focal adhesion kinase)–ROCK (Rho-associated kinase) pathway as well as that mediated by the transcription factors YAP and TAZ have been shown to increase the stiffness of the ECM and thereby to promote the proliferation, epithelial–mesenchymal transition, metastasis, and chemotherapy resistance of PDAC cells, as well as the activation of CAFs [21], with this latter effect leading to increased fibrosis and likely contributing to the physical barrier to effective drug delivery. An abnormal and dysfunctional tumor vasculature also impedes drug delivery to the cancer cells. Targeting of the stroma is thus considered a potential strategy to improve anticancer drug efficacy and patient survival, with numerous such approaches, including vessel normalization, having been pursued [19,20,21,22]. Although clinical trials of agents that target the PDAC vasculature have not revealed a beneficial effect on patient survival, recent studies have suggested the possibility that normalization of the tumor vasculature might promote antitumor immune responses [22].

Similar to PDAC, cholangiocarcinoma is often characterized by a pronounced desmoplastic reaction in tumor tissue. Cholangiocarcinoma is also highly aggressive, is heterogeneous at both intertumoral and intratumoral levels, and has a poor prognosis [23]. The dense stroma of cholangiocarcinoma (including CAFs, immune cells, and the ECM) is thought to contribute to its treatment refractoriness [24,25]. CAFs interact with the cancer cells and play a role in cholangiocarcinoma progression and metastasis. The cancer cells secrete platelet-derived growth factor (PDGF)-D and TGF-β1, both of which activate CAFs, whereas CAFs secrete PDGF-B, heparin-binding EGF-like growth factor (HB-EGF), and stromal cell-derived factor-1 (SDF-1, also known as CXCL12), and thereby promote tumor growth and invasion [26]. CAFs also contribute to fibrogenesis and ECM remodeling, which promote cholangiocarcinoma cell invasion and therapeutic resistance. Furthermore, cholangiocarcinoma tissue expresses high levels of the immune checkpoint molecules PD-L1 and PD-1, as well as CTLA4 and CD80, which contribute to the escape of cancer cells from the immune system and thereby promote tumor growth and metastasis [26]. The extensive stroma of cholangiocarcinoma is also considered to be a potential therapeutic target. Targeting of the TME in association with other treatment modalities, such as cytotoxic chemotherapy or targeted therapy, has received recent attention as a means to achieve synergistic effects on cholangiocarcinoma growth [27]. Such combination therapies targeting cancer cells and their TME may be a promising approach to cholangiocarcinoma treatment.

## 3. Interactions between the TME and Cancer Cells

### 3.1. CAF-Derived Factors and Roles of CAFs in Cancer

Fibroblasts in normal tissue are resting mesenchymal cells embedded in physiological ECM and are activated to facilitate tissue repair and regeneration during wound healing, as well as in association with tissue inflammation and fibrosis [5]. In cancer tissue, fibroblasts are a major component of the tumor stroma of both primary and metastatic tumors. CAFs contribute to cancer through multiple pathways and complex interactions with other cell types in the TME [28,29]. Analogous to the functions of fibroblasts in normal tissue, CAFs play an important role in tumor-promoting inflammation and fibrosis in cancer (the wound that never heals) [5]. CAFs secrete growth factors, inflammatory ligands, and ECM proteins that promote tumor growth, therapy resistance, and immune exclusion [2]. Recent studies have revealed that CAFs and CAF-dependent ECM remodeling are strongly correlated with cancer chemoresistance [30]. CAFs themselves thus act as a physical barrier to the delivery of anticancer drugs and the infiltration of immune cells into tumor tissue [28]. They also produce ECM molecules, including collagen, fibronectin, laminin, and proteoglycans, with the ECM of tumor tissue tending to be denser and stiffer than that of normal tissue. Such CAF-dependent ECM remodeling thus gives rise to desmoplasia and fibrosis in the tumor stroma and generates a physical barrier between cancer cells and therapeutic drugs that promotes chemoresistance [31,32]. In addition to producing ECM components that contribute to the structure and function of the tumor stroma, CAFs undergo epigenetic changes that result in the production of secreted factors, exosomes, and metabolites that influence tumor angiogenesis, immunology, and metabolism [29].

CAF-derived factors, such as matrix metalloproteinase 2 (MMP2), the chemokine CXCL12, TGF-β, and IL-6, facilitate the proliferation and invasion of cancer cells in various tumors [33]. CXCL12 secreted by CAFs positive for α-smooth muscle actin (α-SMA) thus promotes the proliferation of cancer stem cells through interaction with its receptor CXCR4 [34]. CAF-derived CXCL12 also induces M2 macrophage polarization and thereby contributes to cisplatin resistance in colorectal cancer [35]. CAFs also release exosomes that increase chemoresistance in pancreatic cancer [36], whereas tumor-cell-derived exosomal microRNAs (miRNAs) promote lung metastasis of hepatocellular carcinoma through CAF activation [37].

Interleukin-6 released from α-SMA^+^ CAFs induces the expression of programmed cell death-ligand 1 (PD-L1) through activation of a signal transducer and activator of transcription 3 (STAT3) in neutrophils and thereby promotes immunosuppression in the TME of hepatocellular carcinoma [38]. TGF-β released from α-SMA^+^ CAFs also regulates the activity of NK cells [39]. Both TGF-β and IL-6 are implicated in the restraint of dendritic cell function and the maturation and consequent impairment of T-cell activation and induction of T-cell anergy [40,41]. VEGF derived from α-SMA^+^ CAFs also suppresses dendritic cell generation and maturation [42,43]. CAFs positive for both fibroblast activation protein (FAP) and α-SMA have been found to inhibit the proliferation of CD8^+^ cytotoxic T cells, and TGF-β and VEGF derived from cancer cells and CAFs to promote the recruitment of CD4^+^CD25^+^ Tregs, in head and neck squamous cell carcinoma [44].

Some individuals with cancer remain asymptomatic for many years after antitumor therapies, with their residual cancer cells entering a state of quiescence known as cancer dormancy [45]. CAFs play a pivotal role in determination of the ultimate fate of such dormant cancer cells [46,47]. Recent studies have shown that the depletion of CAFs confers a more aggressive phenotype in pancreatic cancer [9,48]. CAFs may influence the dormant state of cancer cells through ECM remodeling or the production of exosomes, TGF-β, interferons, insulin-like growth factor (IGF), fibroblast growth factor (FGF), macrophage colony-stimulating factor (M-CSF), and interleukins [45]. Although it is clear that dormant cancer cells are key to disease relapse in many cancer types, it remains unclear whether, when, and how these cells should be treated [49,50].

Given their multiple tumor-promoting roles, CAFs have long been considered an attractive therapeutic target. Recent research has shown that CAFs are closely associated with the response to immunotherapy in cancer patients, and has implicated biglycan, a CAF-derived proteoglycan in the ECM, in this association [51]. Patients with a high biglycan level in tumor tissue thus showed a worse response to immunotherapy and a smaller number of tumor-infiltrating CD8^+^ T cells than did those with a low biglycan level. This study suggests that biglycan derived from CAFs may be an unfavorable indicator for immunotherapy response and overall survival in cancer patients, and that it is a potential therapeutic target to overcome immunotherapy resistance.

### 3.2. CAFs Contribute to Tumor Heterogeneity in Lung Adenocarcinoma

**Figure 2:**
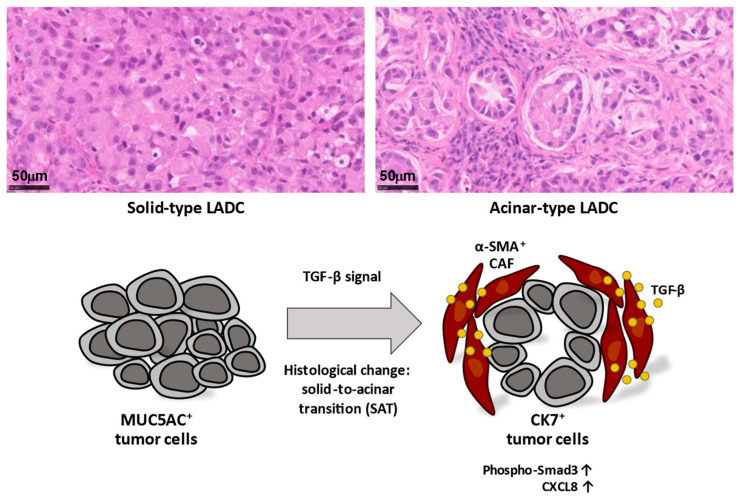
Lung adenocarcinoma (LADC) cells form tumors with distinct histological features, including MUC5AC^+^ solid-type and CK7^+^ acinar-type tumors. Acinar-type tumors manifest glandlike structures encircled by stromal cells, including α-SMA^+^ cancer-associated fibroblasts (CAFs). CAFs secrete TGF-β, and activation of TGF-β-Smad3 signaling induces a histological change termed the solid-to-acinar transition (SAT) in lung cancer cells. Activation of TGF-β signaling also contributes to upregulation of CXCL8 expression in LADC cells, which has the potential to induce changes to the tumor microenvironment (TME). The induction of SAT in cancer cells as well as changes to the TME by CAF-dependent TGF-β signaling may therefore contribute to tumor heterogeneity.

According to the latest World Health Organization classification, invasive lung adenocarcinoma (LADC) is classified histologically as lepidic, acinar, papillary, micropapillary, or solid. Most LADCs manifest several of these histological subtypes and therefore show intratumoral heterogeneity. We recently showed that A549 human LADC cells form tumors with distinct histological features—mucin 5AC (MUC5AC)-expressing solid-type or cytokeratin 7 (CK7)-expressing acinar-type tumors—depending on the site of development in immunodeficient mice, and that α-SMA-positive CAFs surrounding LADC cells induce acinar tissue formation via activation of TGF-β-Smad3 signaling [43]. This TME-induced solid-to-acinar transition (SAT) (Figure 2) was thus characterized by changes in morphology (solid to acinar), gene expression (in particular, expression of *MUC5AC* and *KRT7*), and TGF-β signaling. Immortalized CAFs derived from acinar-type tumors induced SAT in three-dimensional (3D) cocultures with A549 cells. Exogenous TGF-β1 or forced expression of an active form of TGF-β1 increased CK7 expression and attenuated MUC5AC expression in A549 cells. Furthermore, inhibition of TGF-β signaling in A549 cells or knockdown of TGF-β1 in CAFs suppressed acinar tumor formation [43]. These findings thus indicate that CAF-dependent TGF-β signaling determines the histological signature of LADC and thereby contributes to the histological heterogeneity of LADC tissue.

### 3.3. Cancer Cell–CAF Interactions Influence the TME

RNA-sequencing analysis revealed that SAT is associated with angiogenesis and neutrophil recruitment, with the activation of TGF-β signaling in LADC cells induced by interaction with CAFs resulting in upregulation of *CXCL8* expression in the cancer cells [52] (Figure 2). *CXCL8* encodes the chemokine CXCL8, which is also known as IL-8. The expression of CXCL8 was increased in CK7- and phospho-Smad3-positive acinar-type tumor tissue compared with solid-type tumor tissue in the A549 xenograft model, as well as in human LADC tissue and mutant KRAS-driven mouse lung cancer tissue. CXCL8 plays a key role in inflammatory responses, promotes the recruitment of neutrophils into tumor tissue, and serves as a proangiogenic factor. It therefore likely contributes to the neutrophil recruitment and angiogenesis associated with SAT. Both neutrophils and TAMs promote angiogenesis by secreting proangiogenic factors, such as VEGF and tumor necrosis factor-α (TNF-α), as well as IL-8/CXCL8 and various other chemokines [53].

Recent studies have shown that interactions between CAFs and immune cells have the potential to modulate the tumor immune environment and lead to immune suppression in tumor tissue [54]. Neutrophils are a key component of the tumor immune environment, with tumor-infiltrating neutrophils being referred to as tumor-associated neutrophils (TANs). CAFs are able to promote the recruitment of peripheral neutrophils into tumor tissue via the secretion of SDF-1α/CXCL12. In addition, activation of the STAT3 signaling pathway and induction of PD-L1 expression in TANs by CAF-derived IL-6 result in the inhibition of T cells and consequent immune tolerance [38]. IL-8/CXCL8 is also thought to contribute to immune suppression that can underlie resistance to immune checkpoint therapy in cancer patients [55,56]. The secretion of CXCL8 during CAF-induced SAT may therefore elicit tumor immune suppression through dynamic changes in the TME.

## 4. Experimental Models for Investigation of Cancer Cell–TME Interactions and the TME Network

### 4.1. Animal Models

Immunocompetent mouse cancer models that recapitulate the TME are helpful for investigation of the interactions of cancer cells with cells of the TME, including immune cells. We have previously developed organoid-based orthotopic and syngeneic mouse models of LADC [57] and biliary tract cancer [58]. In the case of the former models, we isolated EpCAM-positive epithelial cells from the *Ink4a/Arf*^−/−^ mouse lung and cultured them as organoids to maintain epithelial stem cell properties. These cells were then transformed by KRAS(G12V) or the EML4-ALK fusion oncoprotein and transplanted via the trachea into the lung of syngeneic wild-type mice, where they formed tumors that expressed the lung lineage marker TTF-1 and closely recapitulated the pathology of human LADC [57].

For the mouse models of biliary tract cancer (intrahepatic cholangiocarcinoma, gallbladder cancer, or extrahepatic cholangiocarcinoma), we isolated EpCAM-positive epithelial cells from the mouse intrahepatic bile duct, gallbladder, or extrahepatic bile duct and then established organoids with epithelial stem cell properties derived from these cells. The introduction of KRAS(G12V) and homozygous deletion of *Ink4a/Arf* in the cells of each organoid type conferred the ability to form lethal metastatic adenocarcinoma with CK19-positive differentiated components and a pronounced desmoplastic reaction on cell transplantation into syngeneic mice. The resulting mouse tumors appeared to recapitulate the pathological features of human cholangiocarcinoma [58]. Machine learning analysis of H&E-stained images of tumor tissue revealed similarities between the mouse tumors and human cholangiocarcinoma [59]. In the biliary tract cancer models, CD4^+^ T cells, CD8^+^ T cells, F4/80^+^ macrophages, and CD31^+^ endothelial cells were detected mostly in desmoplastic areas of tumor tissue where cancer cells were surrounded by stromal cells and the ECM [58]. PD-L1-expressing cells were also detected in the tumor tissue [58]. Such immunocompetent mouse cancer models that recapitulate the TME should thus provide a better understanding of the interactions of cancer cells with the TME, including the immune system network.

### 4.2. Three-Dimensional Cell Coculture and Organoid Culture Systems

In vitro 3D coculture and organoid culture are artificial reconstitution systems for recapitulation of the TME. Novel 3D organoid systems based on patient-derived cancer cells and endogenous immune cells (T cells, B cells, NK cells, and macrophages) have recently been developed [60]. These models appear to recapitulate immune checkpoint blockade and should prove useful for investigation of the interactions of cancer cells with cells of their microenvironment in vitro. In addition, patient-derived organoids that reproduce the tumor immune environment might allow personalized immunotherapy testing. Such 3D organoids also allow the investigation of tumor dormancy [61].

Three-dimensional microfluidic organ-on-chip systems recapitulate human organ microenvironments and simulate cell-cell and cell-ECM interactions that occur in vivo [62]. A recent study characterized the development of colorectal cancer on a chip with a system that mimics the intravasation of tumor cells into a blood vessel and allows monitoring of the metastatic process via on-chip imaging and mass-spectrometry-based metabolomics analysis [63].

A technique known as in situ decellularization of tissues (ISDoT) that is able to completely remove cells from whole organs, leaving the ECM in its native state, was recently described [64]. This procedure allows the observation of native ECM structure and composition under normal and cancerous conditions. Similarly, 3D fibroblast-derived matrices in vitro have been found to resemble mesenchymal matrices in vivo [65,66]. Such a matrix derived from lung fibroblasts (the lung being a target organ for metastasis of breast cancer) was recently applied to high-throughput drug screening after reconstitution with breast cancer cells and revealed a drug response profile that differed from that of breast cancer cells cultured on plastic [67]. This finding highlights the importance of taking the ECM into account when testing the drug responses of cancer cells in vitro [67].

In early 2023, the U.S. Food and Drug Administration (FDA) eliminated the requirement that drugs in development undergo testing in animals before being administered to participants in human trials [68]. This change opens the door to an increased role for other approaches—such as computer modeling, 3D coculture and organoid culture, and organ-on-chip systems—both in drug development [69] and as tools to predict the responses of individual patients in personalized medicine [70].

## 5. Potential Strategies for Treatment of Heterogeneous Tumors

### 5.1. Small Molecules

**Figure 3:**
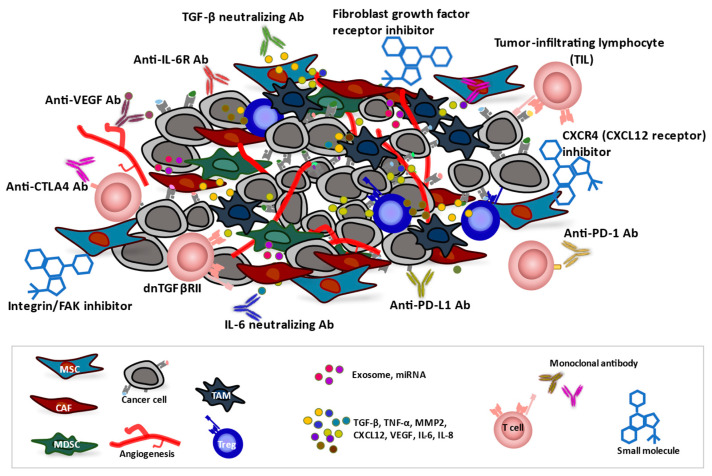
The tumor microenvironment (TME) and anticancer therapeutic approaches. Cancer-associated fibroblasts (CAFs), tumor-associated macrophages (TAMs), myeloid-derived suppressor cells (MDSCs), and mesenchymal stromal cells (MSCs) contribute to generation of a TME that supports the growth of tumor cells as well as renders them poorly immunogenic and resistant to therapies. Infiltrated or induced regulatory T cells (Tregs) in the TME also have a strong immunosuppressive function. CAF-dependent TGF-β signaling induces remodeling of tumor tissue and may promote angiogenesis. CAF-derived factors, including TGF-β, TNF-α, MMP2, CXCL12, VEGF, IL-6, and IL-8, promote the invasion of cancer cells into surrounding tissue, neutrophil recruitment, and angiogenesis. Therapeutic agents, including monoclonal antibodies (Abs), small molecules, and genetically engineered T cells, can influence the TME and promote antitumor immune responses and thereby inhibit tumor growth and survival.

Integrins serve as receptors for various ECM proteins and are widely expressed by malignant and stromal cells of tumors at focal adhesions. Furthermore, FAK, a key mediator of integrin signaling, is upregulated in various cancer types [71,72,73], and inhibition of integrin signaling has been shown to slow tumor progression [74] (Figure 3). Integrins have therefore been investigated as potential therapeutic targets in cancer, with ~30 clinical trials having been initiated to date [75]. In addition, allosteric activation of the leukocyte-specific integrins α_L_β_2_ and α_4_β_1_ in T cells with the small-molecule drug 7HP349 was found to enhance T-cell activation and adhesion and thereby to improve the penetration of T cells into tumors in mouse models of melanoma and colon carcinoma [76,77]. In 2022, the FDA granted fast-track designation for use of 7HP349 in combination with a CTLA4 inhibitor in patients with unresectable or metastatic malignant melanoma after failure of treatment with a PD-L1 inhibitor. FAK plays an important role in the growth, adhesion, invasion, metastasis, and survival of tumor cells, as well as in angiogenesis [72,73], and the FAK inhibitor VS-4718 was found to attenuate ECM remodeling and to increase sensitivity to chemotherapy and immunotherapy in a mouse model of pancreatic cancer [78]. FAK inhibitors also improved the efficacy of various anticancer drugs—including the chemotherapeutic agents gemcitabine, paclitaxel, and carboplatin, the PD-1 inhibitor pembrolizumab, and the kinase inhibitor VS-6766—by overcoming drug resistance [79,80,81].

Nicotinamide adenine dinucleotide phosphate oxidase 4 (NOX4) is implicated in CAF-mediated tumor progression. Pharmacological inhibition of NOX4 with GKT137831 (setanaxib) was found to abrogate CAF-dependent tumor cell migration and invasion and to overcome CAF-mediated resistance to chemotherapy and immunotherapy in xenograft tumor models [82,83,84].

Fibroblast growth factor receptors (FGFRs) are a family of receptor tyrosine kinases that are expressed at the cell surface and whose aberrant expression has been demonstrated in various types of solid tumors. FGFRs regulate key developmental processes—including cell proliferation, survival, and migration—as well as oncogenesis [85]. Multiple small-molecule inhibitors that target this family of kinases have been developed and are currently being tested in preclinical studies as well as in phase 1, 2, and 3 clinical trials. The pan-FGFR inhibitors erdafitinib (JNJ-42756493) and pemigatinib block FGFR phosphorylation and signaling and thereby induce cell death, and they have been approved by the FDA for the treatment of urothelial carcinoma and cholangiocarcinoma, respectively [86] (Figure 3).

CAFs influence the trafficking of lymphocytes by producing the chemokine CXCL12, which binds to both CXCR4 and CXCR7. The CXCL12-CXCR4/CXCR7 axis in the TME plays a pivotal role in tumor development, survival, angiogenesis, and metastasis [87]. In a mouse model of pancreatic cancer, inhibition of CXCR4 resulted in T-cell infiltration into the tumor and conferred a response to anti-PD-L1 antibody administration [88] (Figure 3). A clinical study also showed that the inhibition of CXCR4 with AMD3100 (plerixafor, or Mozobil) in patients with microsatellite-stable (MSS) colorectal cancer or PDAC tumors resistant to immunotherapy promoted an antitumor immune response [89]. The combination of CXCR4 inhibition and T-cell checkpoint antagonism might therefore prove to be beneficial for cancer patients.

### 5.2. Antibodies

In addition to small molecules that inhibit tyrosine kinases, several monoclonal antibodies have also been developed. These agents include FGFR-targeted antibodies that are currently under preclinical or clinical evaluation [90] (Figure 3). Although aprutumab ixadotin (BAY 1187982), an antibody-drug conjugate that targets FGFR2, was found to inhibit tumor growth in xenograft models of gastric and breast cancer [90], it did not induce a favorable clinical response in a human trial [91].

The immunomodulatory cytokine IL-6 is a key regulator of immune cell polarization and is implicated in cancer progression and the response to chemotherapy [92,93,94,95]. IL-6 released by CAFs has been found to be associated with malignancy [96]. A monoclonal antibody to the IL-6 receptor (tocilizumab) abrogated CAF-dependent tumor growth and resistance to radiotherapy to similar extents as did a STAT3 inhibitor or a neutralizing antibody to IL-6 in mouse models of breast cancer [3] (Figure 3). Similarly, blockade of the IL-6 receptor (IL-6R) on cholangiocarcinoma cells was found to enhance sensitivity to the chemotherapeutic drug gemcitabine [97].

The regulation of vascular growth by CAFs contributes to tumor progression [98]. Antiangiogenic agents that target VEGF (including the monoclonal antibody bevacizumab) or its receptors (including sorafenib and sunitinib) have shown antitumor as well as antiangiogenic activity [99,100,101,102,103] (Figure 3). Although some studies suggest that antiangiogenic therapy alone has limited benefits for cancer treatment [99,104,105,106], it might induce tumor dormancy and thereby delay time to progression. Combination treatment with antiangiogenic agents and either radiation, chemotherapy, or immunotherapy may provide a better antitumor efficacy [107,108].

### 5.3. T Cells

Adoptive cell therapy has emerged as a promising cancer immunotherapy. Such therapy with tumor-infiltrating lymphocytes (TILs) or with T cells expressing genetically manipulated T-cell receptors (TCRs) or chimeric antigen receptors (CARs) aims to modify the immune system so that it recognizes tumor cells and mediates an effective antitumor response in the TME [109] (Figure 3). Since their initial identification in 1986 [110], TILs have been widely studied as a potential basis for personalized cancer treatment, with favorable results having been obtained for solid tumors. TILs are isolated from tumor tissue of the patient and expanded in vitro on the basis that they are likely to be highly tumor-reactive and neoantigen-specific T cells. In 2021, Iovance successfully completed a phase 2 trial of a TIL product, Lifileucel (LN-144), for patients with advanced melanoma whose disease had progressed during treatment with immune checkpoint inhibitors. The study revealed an objective response rate of 36%, including 2 complete responses and 22 partial responses among 66 patients, and a disease control rate of 80% [13,111].

The TGF-β signaling pathway plays an important role in the promotion of metastasis and angiogenesis, as well as in immune suppression in the TME. In particular, TGF-β inhibits the proliferation of T cells, their differentiation into helper T cells and cytotoxic T cells, and their activation by antigen-presenting cells [112,113,114]. Inactivation of the endogenous TGF-β type II receptor (TGFβRII) was found to render cells resistant to TGF-β [115,116]. Expression of a dominant negative form of TGFβRII (dnTGFβRII) that lacks the intracellular domain necessary for downstream signaling but which still binds TGF-β was thus shown to block TGF-β signaling in T cells and to increase their ability to infiltrate, proliferate, and mediate an antitumor response in the TME [117] (Figure 3). A transgenic mouse model in which dnTGFβRII is expressed specifically in T lymphocytes revealed that the mutant protein promoted T-cell proliferation and differentiation, increased the secretion of granzyme A, granzyme B, perforin, and interferon-γ by the cells, enhanced their cytotoxicity and antitumor effects, and reduced the proportion of Tregs in tumor tissue and the spleen of tumor-bearing mice [112]. T cells have also been engineered to express both dnTGFβRII and a CAR construct that recognizes prostate-specific membrane antigen (PSMA). The resulting cells manifested increased proliferation, cytokine secretion, resistance to exhaustion, long-term persistence in vivo, and tumor eradication in a mouse model of human aggressive prostate cancer [117]. A phase 1 clinical trial of such modified T cells (NCT03089203) demonstrated their feasibility and safety for the treatment of metastatic castration-resistant prostate cancer patients [118]. Although further studies are necessary, the clinical application of dnTGFβRII may prove to be an effective approach to targeting of the TME in cancer patients.

### 5.4. Molecularly Targeted Cancer Therapies Affect the TME

Driver mutations such as activating mutations of the EGF receptor gene (*EGFR*) not only enhance the proliferation and survival of cancer cells but also influence their immune environment. In particular, *EGFR* mutation-positive lung cancer cells generate an immunosuppressive microenvironment by secreting chemokines that attract Tregs. Treatment with an EGFR inhibitor thus enhanced the antitumor effect of immune checkpoint inhibitors in a mouse model of *EGFR*-mutated lung cancer [119]. The potential of EGFR inhibitors as immunotherapy sensitizers was also supported by a study with an experimental model of PDAC [120]. Moreover, a small-molecule inhibitor of mutant KRAS that induced tumor regression in an immunocompetent mouse model of PDAC [121] was found not only to trigger tumor cell proliferative arrest and apoptosis but also to elicit changes to the TME, including effects on fibroblasts, the ECM, and macrophages. Of note, T cells were necessary for the maximal antitumor effect of the inhibitor, with the depletion of T cells being found to promote tumor regrowth after therapy [121].

Immunocompetent mouse models have thus revealed that the antitumor action of molecularly targeted agents is mediated in part through effects on the tumor immune environment that may sensitize cancer cells to immunotherapy. Although immunotherapy has revolutionized cancer care, some patients do not respond to such therapy as a result of poor infiltration and activation of T cells in the TME. Combination regimens of RAS or EGFR inhibitors and immunotherapeutic agents have the potential to improve the therapeutic responses of cancer patients.

## 6. Conclusions

Cancer cells interact with surrounding stromal components in tumor tissue. Recent studies have shown that interactions of cancer cells with the TME result in tumor immune suppression. Combinations of cancer-cell-targeted therapies and TME-targeted therapies are thought to have the potential to improve the survival of cancer patients. The TME is complex, heterogeneous, and highly dynamic with regard to its composition. Studies of cancer models that recapitulate human tumor tissue are thus required to provide insight into this environment. Immunocompetent mouse cancer models should also help to understand TME–cancer cell interactions and the immune network in tumor tissue, as well as provide a basis for the development of new therapeutic strategies.

## Data Availability

Since no new data were created in this study, data sharing is not applicable to this article.
